# Simulation Modifies Prehension: Evidence for a Conjoined Representation of the Graspable Features of an Object and the Action of Grasping It

**DOI:** 10.1371/journal.pone.0000311

**Published:** 2007-03-21

**Authors:** Victor Frak, Isabelle Croteau, Daniel Bourbonnais, Christian Duval, Cyril Duclos, Henri Cohen

**Affiliations:** 1 Département de kinanthropologie, Université du Québec à Montréal, Montreal, Quebec, Canada; 2 Institut de Réadaptation de Montréal, Centre de recherche interdisciplinaire en réadaptation du Montréal métropolitain, Université de Montréal, Montreal, Quebec, Canada; 3 Psychology and Cognitive Neuroscience Laboratory (CNRS-Paris Descartes), Quebec Memory and Motor Skill Disorders Research Centre, Clinique Sainte-Anne, Québec City, Quebec, Canada; University of Birmingham, United Kingdom

## Abstract

Movement formulas, engrams, kinesthetic images and internal models of the body in action are notions derived mostly from clinical observations of brain-damaged subjects. They also suggest that the prehensile geometry of an object is integrated in the neural circuits and includes the object's graspable characteristics as well as its semantic properties. In order to determine whether there is a conjoined representation of the graspable characteristics of an object in relation to the actual grasping, it is necessary to separate the graspable (low-level) from the semantic (high-level) properties of the object. Right-handed subjects were asked to grasp and lift a smooth 300-g cylinder with one hand, before and after judging the level of difficulty of a “grasping for pouring” action, involving a smaller cylinder and using the opposite hand. The results showed that simulated grasps with the right hand exert a direct influence on actual motor acts with the left hand. These observations add to the evidence that there is a conjoined representation of the graspable characteristics of the object and the biomechanical constraints of the arm.

## Introduction

Grasping is a kind of active contact with objects in the environment. This action requires a number of visuomotor transformations to code for the intrinsic and extrinsic properties of the objects to be grasped (i.e., shape and location, respectively). The geometric attributes of the object will trigger the finger grasp, while its semantic properties will determine functional interactions. These attributes and properties are not mutually exclusive and the pragmatic and semantic modes of operation with objects interact. The distinction between these properties has been made evident in lesion studies in humans [1]. These observations suggest that actions have a central origin and that kinesthetic images formed from sensory clues are stored in the motor cortex [2]–[Bibr pone.0000311-Liepmann1] [Bibr pone.0000311-Kleist1] [5]. While the superior parietal lobule is involved in the automatic control of visually guided actions, the inferior parietal lobule (particularly on the left side) is concerned with the planning of actions and involves the retrieval of complex representations thought to be produced in that structure [6]. The parietal areas, together with the premotor cortex [7], account for so-called pragmatic representations.

The relevance of the pragmatic aspects of prehension in normal behavior is an important question. Interestingly, Sakata et al. [8] have described neuronal discharges in the monkey in response to both passive and active observation of objects with graspable shapes (e.g., cylinder, cube). This neuronal activity has also been taken as evidence of the anatomofunctional substrate of a pragmatic theory of grasping in humans [9]. It remains to be determined whether this relationship between the internal representations of the graspable characteristics of the object and of grasping that object is a property seen in normal subjects.

Orientation grasping [10] is the appropriate paradigm to investigate this question. In an earlier study, we showed that precision grasping with one hand influences grasping orientation, in an anticlockwise manner, with the other hand [11]. It is likely that this result reflects the existence of a functional engram combining the graspable characteristics of the cylinder and the grasp orientation.

Grasp orientation, defined by the opposition axis (OA), represents the effector of the movement and is a main parameter to control for when completing a grasp [12]. Paulignan et al. [13] analyzed the OA during the grasping of cylinders of various sizes and weights placed in different locations. They found that the OA orientation was constant with respect to an egocentric frame of reference for all conditions. Thus, it is reasonable to think that in these circumstances the OA was computed from representational grasping coordinates. Nevertheless, in view of the fact that perception and action go hand in hand in motor activities [14], it is difficult to dissociate somesthetic afferences from grasp representation when healthy subjects produce a real grasp.

In order to determine whether the graspable characteristics of an object and the associated grasping orientation have a conjoined representation, it is necessary to separate the object's geometric properties from its semantic properties. In this study, we asked subjects to grasp and lift a 300-g smooth cylinder with one hand, before and after judging the feasibility of a “grasping for pouring” action with a considerably smaller cylinder, using the other hand. What the actual grasp and the simulated grasp share in common is the graspable characteristics of the object.

## Methods

### Participants

Twenty-one healthy right-handed volunteers participated in the experiment (age range between 21 and 52 years, mean = 26.6 years; 5 women, 16 men). Handedness was assessed using the Edinburgh Handedness Inventory [15]. Only subjects scoring a laterality quotient of 100 were included in the study. All participants were recruited and tested in accordance with the ethical considerations set out by the Centre for Interdisciplinary Research in Rehabilitation of Montreal's ethics committee. Subjects were initially instructed about the methods used in the study; the purpose of the study was revealed once the experiment was over.

### Procedure

Participants were seated in front of a table; the initial position of the left hand was 13 cm left of the sagittal axis, while the right hand was 13 cm to the right. Participants were asked to perform 10 consecutive real grasps with one hand before and after 400 simulated grasps with the other hand. For the real task condition, they were asked to reach for, grasp, lift and return to its original position a smooth 300-g resin cylinder (6 cm in diameter, 10 cm high) placed in the center of the table at a distance of 32 cm from the body plane, using a precision grip formed by the thumb and the index finger only. The opposition axis (OA) was defined as the line connecting these two contact points on the cylinder [10]. Ten subjects performed the real grasps with their left hand, and 11 with their right; the tips of their thumbs and index fingers were painted in order to mark the cylinder. Subjects were always presented with a clean cylinder as the contact points were cleaned before each grasp. The OA was measured with respect to the horizontal plane by means of a protractor and was the dependent variable in this task.

**Figure 1:**
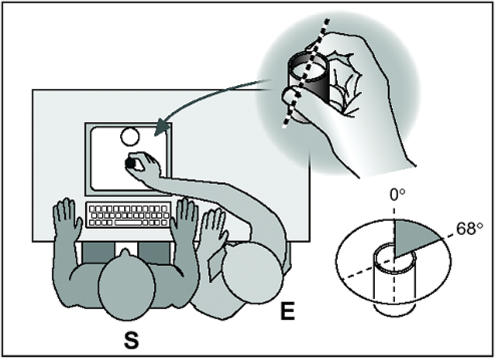
Observed task for grasps performed with the right hand. Subjects (S) were seated in front of a 15″ monitor lying flat with the screen perpendicular to the body axis. The experimenter (E) lifted the plastic cylinder filled with water, poured the water into another container and returned the cylinder to its original position using a precision grip formed by the right thumb and index fingers. The preferred orientation of the real OA used by the experimenter was from 0° to 68°.

In the simulated grasp condition, participants were seated in front of a 15″ monitor, which was lying flat with the screen perpendicular to the body axis and at a distance of 45 cm under the orbitomeatal line. The experimenter first performed five consecutive precision grasps for pouring (grasping, lifting, and pouring) using an opaque cylindrical container filled with water (3 cm in diameter, 5 cm high, weight 30 g) placed in the center of the monitor screen, 32 cm in front of the subjects. The experimenter used his right hand to grasp the container with subjects who were required to simulate a grasp with their right hand, and his left hand for subjects simulating a grasp with their left hand. The OA orientation used by the experimenter was between 0° and 68° with respect to the horizontal plane with the right hand and between −34° and −68° with the left hand. Figure 1 shows the subject's and experimenter's positions for grasps performed with the right hand.

**Figure 2:**
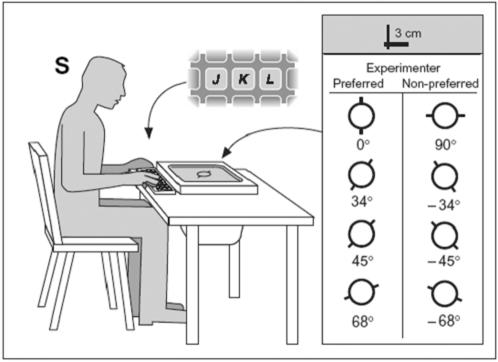
Simulated task with the right hand. Subjects (S) were asked to judge as quickly as possible whether the previously observed action of grasping the cylinder full of water and emptying it into the other container would be possible with the fingers placed according to the opposition axis indicated on the circle. The subjects were asked to rate the level of feasibility of the grasp by pressing keyboard keys (j ,easy; k, difficult; l, impossible).

Subsequently, the objects were removed from the monitor surface and, following a brief training period, the simulation task proper was initiated. For each trial, a central fixation point, presented for 500 ms, was followed by an image of the upper surface of a cylinder (a circle) which remained on the screen, in the same location where the real cylinder was placed during the preliminary run, until a response was given. Each circle was marked with two contact points which defined OAs at −68°, −45°, −34°, 0°, 34°, 45°, 68° and 90° from an egocentric reference frame (see Figure 2). The subjects' task consisted in judging as quickly as possible whether the previously observed action of grasping a cylinder full of water and emptying it into the other container would be possible with the fingers placed on the opposition axis indicated on the circle. No actual grasp was allowed. The subjects had to rate the level of feasibility of the grasp by pressing keyboard keys (right hand simulations: j, easy; k, difficult; l, impossible; reverse order for left hand simulations) with the three middle fingers. Eight orientations were randomly displayed 50 times each. Feasibility level and response time were recorded. Figure 2 shows a simulated grasp trial with the right hand.

### Data analysis

It has been demonstrated that the preferred grasping orientation from the first-and in the third-person perspectives is similar [16]. The eight different OA orientations presented during the simulated task were grouped into two clusters: experimenter-preferred angles (for the right hand: 0°, 34°, 45°, 68°; for the left hand: −34°, −45°, −68°) and non-preferred angles (right hand: 90°, −68°, −45°, −34°; left hand: 0°, 34°, 45°, 68°, 90°).

Feasibility levels (easy, difficult, impossible) and response times were compared for these clusters of preferred and non-preferred orientations in separate three-way ANOVAs.

T-tests for dependent samples were also used to compare OA orientation in the two groups of subjects, before and after the simulated task.

The significance level was set at *p*<.05.

## Results

### Simulated grasp condition

**Figure 3:**
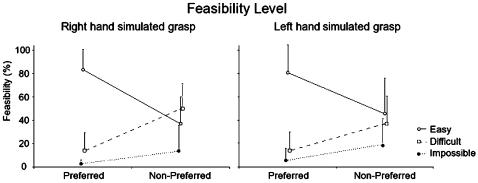
Simulated task: Feasibility level. The performance of subjects in judging whether a particular grasp is easy or difficult shows that there is a good relationship between preferred OA orientation in real and simulated movements. Thus, for all subjects simulation of grasping movements was at the level of the central representation of action.

A significant main effect of orientation on feasibility level (F_(1,20)_ = 34.1, p<.001) and on response time (F_(1,20)_ = 47.1, *p*<.001) was revealed. The participants who simulated a right-hand grasp judged the preferred angles to be easy in 83.5% of the trials. In contrast, the non-preferred angles were judged to be difficult or impossible in 63.3% of cases (*p*<.003) (Figure 3). Response times were also longer with non-preferred (1640±308 ms) than with preferred OA orientations (1333±261 ms; *p*<.001).

The participants who simulated a left-hand grasp judged preferred OA orientations to be easy in 80.8% of the trials, while non-preferred angles were judged to be difficult or impossible in 54.5% of cases. Response times were also longer for non-preferred angles (1925.7±219.0 ms) than for preferred orientations (1715.9±333.8 ms; t = 3.98, *p* = .02).

The results also revealed that simulation of a movement with the left hand (1965±374 ms) takes longer than simulation with the right hand (1491.6±338.1 ms, p<.001), in agreement with previous observations by Parsons et al. [17: p. 6545].

### Real grasp condition

**Figure 4:**
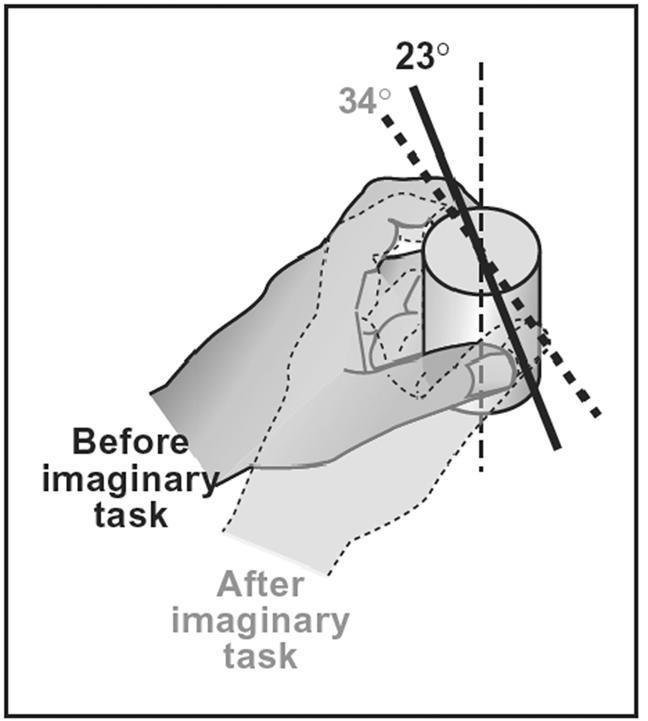
Actual grasping task. Mean OA orientation from an egocentric frame of reference for left hand movements was 23° (ranging from 3° to 31°) before observed and simulated tasks and 34° (ranging from 12° to 44°) after observed and simulated tasks (p<0.0008).

Mean OA orientation from an egocentric frame of reference for left-hand grasps was 23° (range: 3° to 31°) before the simulated grasping task with the right hand, and 34° (range: 12° to 44°) following the simulated grasps. Thus, the grasp orientations after the simulation differed considerably from those before the simulation (p<.0008). The mean OA orientation from an egocentric frame of reference for left-hand movements is shown in Figure 4. Mean OA orientation for right hand grasps before the simulated task with the left hand was 19.8° (range: 11° to 34°), and 21.6° (range: 11° to 38°) following the simulated grasps. Thus, there was no significant difference in right-hand grasping orientations before and after simulations with the left hand (p = .431).

## Discussion

As seen in previous studies [18]–[Bibr pone.0000311-Johnson1] [20], response times increased with the perceived difficulty of the grasp. Thus, imaging of grasping movements took place at the level of the central representation of action. To determine whether a grasp is feasible in the OA task, there is no need for a visual rotation of the object; what is required is a simulation of the grasping movement. Whereas visual shapes can be rotated freely in any direction, the simulation of OA movements allows us to isolate the modality-specific nature of motor imagery [20]–[Bibr pone.0000311-Frak3] [22].

We have established here that motor simulation of the grasping motion of one hand can change the grasping action of the opposite hand. Moreover, the changes in OA orientation observed during actual grasping followed an anticlockwise motion within an egocentric frame of reference, as the subjects' and objects' positions remained constant during the experiment. A similar phenomenon has been reported with real movements during unimanual alternate grasps [11]. In the present study, we show that this change in OA orientation can also occur with simulated grasps, even though the volume of the cylinder and the purpose of the movements differed from the situation for the real movements (i.e., grasping to pour vs. grasping to lift). This implies that the change in OA orientation is under the control of a neural substrate that is strictly dependent neither on actual sensory clues resulting from the motor act itself nor on the purpose of the action. Rather, this modification results from a pragmatic system that codes for the graspable characteristics of the object.

It is well known that motor action of a limb can be influenced by earlier motor acts made by the opposite limb [23]. In addition, it has been demonstrated that imagined motor action of a particular limb can influence the actual motor action of that same limb [24]. Until now, it was not known whether simulated movements of one limb could influence movements of the opposite limb. This is especially relevant since there is ample evidence of bilateral neural network involvement during unimanual movements [25]. If it is true that bilateral regions are available for the planning and execution of unimanual movements, and that a limb may be affected by movements performed by the opposite limb, it seems likely that the bilateral networks used to perform unimanual movements are available for interlimb transfer following simulated movements.

To date, there is good support for bilateral central nervous system (CNS) involvement during unimanual motor acts. For instance, activity in M1 is readily found in electrophysiological recordings in primates during ipsilateral movements of the hand or arm [26], [27]. This activity, however, will not necessarily result in actual movement, probably as the consequence of inhibitory interneurons activated by extensive transcortical inputs [28], [29]. Nonetheless, manifestations of ipsilateral activity can be found during unimanual tasks; surface electromyography recordings have shown clear activity in the homologous muscles during fast repetitive movements [30]. This “motor irradiation” is one indication that the motor system is wired to allow simultaneous movements of homologous muscles, even though these movements are often inhibited. Studies using transcranial magnetic stimulation have also shown that evoked responses of homologous muscles are facilitated by the tonic contraction of the opposite limb [31], and some of the most compelling evidence of active involvement of ipsilateral CNS structures during unimanual movements comes from imaging studies. For example, it has been shown that the level of involvement of ipsilateral cortical structures is related to the movement rate of the limb [32]. Furthermore, it has also been shown that ipsilateral muscles active in the transport grasp mechanism can be influenced by bilateral hemispheric networks in both monkeys [33] and humans [34]. Suspected neural pathways implicated in bilateral control of unimanual movements may include uncrossed corticofugal fibers, branched bilateral corticomotoneuronal projections, intercortical interactions, including bilateral involvement of the primary motor cortex, supplementary motor areas, and the cerebellum and basal ganglia [25]. This bilateral involvement during unimanual tasks may also be manifested in simulated motor acts.
